# Comparison of Essential Oils of *Houttuynia cordata* Thunb. from Different Processing Methods and Harvest Seasons Based on GC-MS and Chemometric Analysis

**DOI:** 10.1155/2021/8324169

**Published:** 2021-07-16

**Authors:** Xue Pan, Haiying Li, Dingfang Chen, Jinjin Zheng, Longhua Yin, Juan Zou, Yalun Zhang, Kaiwen Deng, Meifeng Xiao, Lei Meng, Fuyuan He

**Affiliations:** ^1^College of Pharmacy, Hunan University of Chinese Medicine, Changsha 410208, China; ^2^Hunan Provincial Key Laboratory of Drugability and Preparation Modification of TCM, Changsha 410208, China; ^3^Supramolecular Mechanism and Mathematic-Physics Characterization for Chinese Materia Medica, Hunan University of Chinese Medicine, Changsha 410208, China; ^4^The First Affinity Hospital, Hunan University of Chinese Medicine, Changsha 410007, China

## Abstract

Houttuyniae Herba (HH) refers to the dried aerial part of *Houttuynia cordata* Thunb. (DHC) or the fresh whole grass of *Houttuynia cordata* Thunb. (FHC), where DHC are harvested in summer and FHC around the year. However, harvest seasons and processing methods (i.e., medicinal parts and drying process) might affect the quality of HH. To compare the essential oils (EOs) of DHC and FHC and their two harvest seasons, GC-MS analysis combined with chemometric analysis was applied. The results showed that the oil yield of FHC (0.076 ± 0.030%) was higher than that of DHC (0.038 ± 0.029%), and oil yield was higher in summer than in autumn (0.044 ± 0.029% for DHC1, 0.036 ± 0.028% for DHC2, 0.084 ± 0.026% for FHC1, and 0.067 ± 0.033% for FHC2, respectively). Moreover, hierarchical cluster analysis (HCA) and principal component analysis (PCA) successfully distinguished the chemical constituents of DHC and FHC oils. Additionally, according to orthogonal partial least squares discriminant analysis (OPLS-DA), eleven components were selected as chemical markers for discriminating DHC and FHC, and two and four chemical markers for discriminating two harvest seasons of DHC and FHC, respectively. Among these markers, the average contents of *α*-pinene, limonene, *β*-phellandrene, *α*-terpineol, 4-tridecanone, and ethyl decanoate were higher in FHC oils. In contrast, the average contents of nonanal, 1-nonanol, *β*-cyclocitral, *n*-hexadecanoic acid, and octadecanol were higher in DHC oils. Additionally, the contents of 4-tridecanone and ethyl decanoate were both higher in DHC1 oils than in DHC2 oils. Moreover, the contents of *β*-myrcene and *β*-phellandrene were higher in FHC1 oils, while the contents of 2,6-octadien-1-ol, 3,7-dimethyl-, acetate, and (z)-phytol were higher in FHC2 oils. For these reasons, this study provides a scientific basis for quality control and clinical medication.

## 1. Introduction

*Houttuynia cordata* Thunb. (HC), a promising herbal medicine of the family Saururaceae, is mainly distributed in Asia, including China, India, Thailand, Japan, and Korea. It has a long history of clinical use in traditional Chinese medicine for its several functions, including antifebrile and detoxification, eliminating carbuncle and discharging pus, and promoting diuresis and relieving stranguria [1]. HC mainly contains essential oil (EO), organic acids, flavonoids, phenols, polysaccharides, and alkaloids. EO is one of the main effective parts of HC because it has a variety of pharmacological activities, such as antibacterial, anti-inflammatory, anticancer, antifungal, antiviral, antihistamine, and antidiabetic activities [1–3]. Thus, investigating the chemical compositions of EO could be an effective approach to evaluate the quality of HC. To date, many researchers studied the chemical constituents of EO of HC. Some research teams revealed the main effective components of EO of HC were 2-undecanone, decanal, decanoyl acetaldehyde, *α*-pinene, camphene, *β*-pinene, *β*-myrcene, lauraldehyde, bornyl acetate, limonene, caryophyllene, 4-terpineol, nonanol, and caryophyllene oxide [3]. In addition, some investigators suggested that medicinal parts, production areas, harvest seasons, and processing methods might influence the chemical composition, which in turn affect the therapeutic efficacy of the herbs [1, 3–5].

According to the 2020 edition of Chinese pharmacopoeia, DHC and FHC are indiscriminately used as one herb medicine called Yuxingcao in Chinese (Houttuyniae Herba), and DHC are harvested in summer and FHC around the year. The different processing methods (i.e., medicinal parts and drying process) and harvest seasons might influence their chemical composition and quality. As DHC and FHC are indiscriminately used in clinic and EO is regarded as one of the main effective parts [1], it is necessary to compare the EOs of DHC and FHC and their two harvest seasons systematically and comprehensively. GC-MS fingerprinting technology is the routine method to analyze EO compounds of HC. However, according to our knowledge, only one study directly compared the content differences of 16 components. The authors found 5 components with obvious differences of 2 batches of DHC and 8 batches of FHC from Emei in China [1]. This intuitive comparison could find some components with obvious differences. However, conventional mutual chemical comparison cannot determine elements that result in quality variance. In recent years, chemical fingerprints combined with chemometric analysis such as HCA, PCA, and OPLS-DA have become powerful tools for the identification and quality assessment of traditional Chinese medicine [6–8].

In this study, GC-MS analysis combined with chemometric methods was first applied to compare EOs of DHC and FHC and their two harvest seasons systematically. HCA and PCA were utilized for the classification and distinction of their EOs. Then, OPLS-DA was further employed to identify the potential chemical markers responsible for the discrimination. In addition, the relative content difference of these chemical markers in DHC and FHC and their two harvest seasons were analyzed, and their pharmacological properties were considered. The aim of this study is to investigate the differences of EOs of DHC and FHC and their two harvest seasons and to provide scientific basis for quality control and clinical medication.

## 2. Materials and Methods

### 2.1. Plant Material and Isolation of Essential Oils

Twenty-one batches of DHC and twenty-three batches of FHC were collected from the main production areas in China such as Zhejiang, Guangxi, Jiangxi, Hunan, Guizhou, Sichuan, and Jiangsu, at two harvest seasons (summer season and autumn season). DHC were obtained from the aerial parts of fresh HC, which were dried naturally until the weight was unchanged, while FHC were obtained from the whole grass of fresh HC. The plants were identified by Professor Xiaojiang Zhou (School of pharmacy, Hunan University of Chinese Medicine, Changsha, China). Among all the samples, DHC were labeled as DHC (batches in summer labeled DHC1-1 to DHC1-11 and batches in autumn labeled DHC2-1 to DHC2-10), while FHC were labeled as FHC (batches in summer labeled FHC1-1 to FHC1-12 and batches in autumn labeled FHC2-1 to FHC2-11).

DHC (120 g) or FHC (600 g) were cut into small segments (2–3 cm) and subjected to hydrodistillation in a Clevenger-type apparatus for four hours for isolation of EOs. The oil yields (%, v/w) were calculated in milliliters of oil per 100 g of FHC and the aerial parts of fresh HC for preparing DHC. After being dehydrated over anhydrous sodium sulphate, it was stored at approximately 4°C, ready for GC-MS detection. Each EO was diluted 20 times with n-hexane before GC-MS analysis.

### 2.2. GC-MS Analysis

GC-MS analysis was performed on GC-MS-QP2010 (Shimadzu, Japan) equipped with the DB-5 capillary column (60 m × 250 *μ*m × 0.25 *μ*m film thickness; Agilent). A volume of 1 *μ*L of each sample was injected. The carrier gas was He (purity > 99.999%) at 1.5 mL/min with a split ratio of 10 : 1 and the injector temperature was 250°C. The oven temperature was set at 80°C for 2 minutes and then increased to 125°C at a rate of 10°C/min and maintained for 5 min, to 165°C at a rate of 10°C/min and held for 10 min, to 185°C at a rate of 2°C/min, and finally to 240°C at a rate of 10°C/min and lasted for 20 min. The mass spectrometer was operated in electron impact (EI) mode at 70 eV in a scan range of 35–500 m/z. The temperature of the ion source and transmission line was 250°C and 280°C, respectively. The solvent delay was 4 min.

### 2.3. Data Preprocessing

Peaks were picked up with the signal-to-noise ratio (S/N) > 6. Raw data of GC-MS were transformed into MZXML format by the Shimadzu postrun workstation and finally transformed into ABF format and then processed by Mass Spectrometry-Data Independent Analysis (MS-DIAL) software to detect volatile compounds features and align all the peaks. The components of EO were identified and screened by the MS-DIAL and National Institute of Standards and Technology (NIST) 17.0 Mass Spectra Database. The semiquantitative analysis of volatile compounds was performed by comparing their peak areas in the GC-MS total ion chromatogram. The percentage compositions of common compounds were calculated by the area normalization method.

### 2.4. Statistical Analysis

Heatmap and HCA were generated on the MetaboAnalyst5.0 (https://www.metaboanalyst.ca/) to display the overall difference of chemical compositions of DHC and FHC. PCA and OPLS-DA were applied to investigate the distinction of chemical compositions of DHC and FHC by SIMCA-P 15.0 (Umetrics AB, Umea, Sweden). The significant chemical markers were evaluated based on their *p* value and differing variable importance in projection (VIP) value calculated with OPLS-DA. One-way ANOVA was performed using SPSS 16.0 (Chicago, IL, USA).

## 3. Results and Discussion

### 3.1. EO Yields of DHC and FHC and Their Two Harvest Seasons

**Figure 1:**
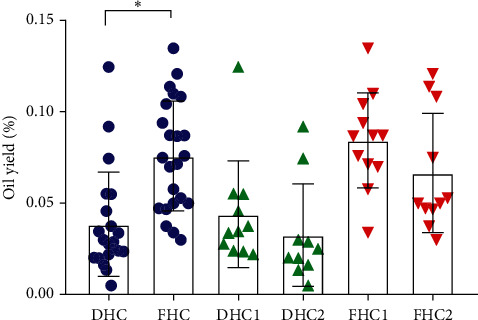
The EO yields (%, v/w) of twenty-one batches of DHC and twenty-three batches of FHC and their two harvest seasons (DHC1 and DHC2; FHC1 and FHC2). Data were presented as mean ± SD. ^*∗*^*p* < 0.05, DHC compared with FHC.

The EO yields (%, v/w) of DHC and FHC and their two harvest seasons (DHC1 and DHC2; FHC1 and FHC2) are shown in Figure 1. The results showed that the EO yield of DHC (0.038 ± 0.029%) was significantly lower than that of FHC (0.076 ± 0.030%) (*p* < 0.05). This was probably due to the loss or decomposition of various volatile components during the drying process [1]. On the other hand, between the two harvest seasons, the EO yields of DHC1 (0.044 ± 0.029%) were a little higher than that of DHC2 (0.036 ± 0.028%), and so were FHC1 (0.084 ± 0.026%) and FHC2 (0.067 ± 0.033%), indicating that the content of EO was higher in summer than that in autumn. One potential explanation for this finding is that the flowering season of HC was in summer and flowers could produce the most oil [9].

### 3.2. Characterization and Classification of Chemical Profiles by GC-MS

**Figure 2:**
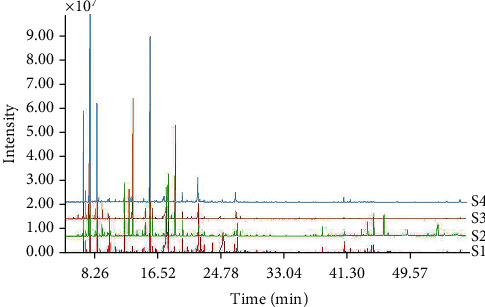
Representative total ion chromatograms of EOs of DHC1, DHC2, FHC1, and FHC2. S1: DHC1; S2: DHC2; S3: FHC1; S4: FHC2.

The EOs extracted from 44 samples of DHC and FHC at two harvest seasons were comprehensively analyzed by GC-MS. According to total ion chromatograms (TIC), the chemical components of DHC and FHC were similar in general but still had some differences. Visually, the quantity and content of peaks of DHC from 42 min to 47 min were higher than of FHC (Figure 2). The chemical constituents of EOs of samples were characterized according to the MS-DIAL and NIST17.0 Mass Spectra Database. Altogether, 69 common constituents were identified, as listed in Table 1. The oils were predominantly composed of two categories of chemical constituents, i.e., nonterpene compounds (aliphatic compounds and aromatic compounds) and terpenoids (terpene hydrocarbons including monoterpene hydrocarbons, sesquiterpene hydrocarbons, diterpene hydrocarbons, and oxygenated terpenoids). The average percentage of the terpenoids and nonterpene compounds were almost the same in both DHC and FHC, in which the content of aliphatic compounds and monoterpene hydrocarbons were present in relatively high amounts (more than 50% and 30%, respectively). The results were consistent with the previous reports [3, 10]. Moreover, the contents of the three most abundant components (2-undecanone, *β*-myrcene, and *β*-pinene) account for about 70% in both DHC and FHC and in the two harvest seasons of DHC and FHC. Besides, 2-undecanone was the most abundant component, which was the quality control component of HC in Chinese Pharmacopoeia.

However, it was worth noting that oxygenated terpenoids in DHC (9.72%) were higher than in FHC (6.55%), whereas terpenoids in DHC (31.95%) were lower than in FHC (35.79%). One potential explanation is the loss of the terpenoids or its oxidation to oxygenated terpenoids during the drying process [11, 12]. Moreover, the content of some chemical constituents had intuitive differences and varied with DHC and FHC and two harvest seasons. The contents of the components such as *α*-pinene, (1S)-(-)-*β*-pinene, *β*-myrcene,(E)-3,7-dimethyl-2,6-octadien-1-ol, and *β*-phellandrene (2.18 ± 1.15%, 1.64 ± 1.79%, 18.36 ± 7.64 ,2.44 ± 1.99%, and 1.82 ± 2.23%, respectively) were higher in FHC oil than in DHC oil (0.63 ± 0.44%, 0.77 ± 1.05%, 17.68 ± 12.99%,1.55 ± 1.73%, and 0.53 ± 1.51%, respectively), while the contents of 1-nonanol, geranyl acetate,2-tridecanone, tetradecanal, and bornyl acetate were higher in DHC oil (3.10 ± 1.50%,2.65 ± 1.80%, 2.29 ± 2.16%, 1.48 ± 1.19%, and 1.89 ± 0.92%, respectively) than in FHC oil (0.49 ± 0.55%, 0.75 ± 0.46%, 1.18 ± 1.10%, 0.82 ± 0.38%, and 1.28 ± 0.26%, respectively). Compared with the results of DHC and FHC, the differences of oils between two harvest seasons (DHC1 and DHC2; FHC1 and FHC2) were not so obvious. Some constituents such as (E)-3,7-dimethyl-2,6-octadien-1-ol and *β*-phellandrene in FHC1 oil (3.04 ± 2.44% and 2.82 ± 2.65%, respectively) were higher than in FHC2 oil (1.80 ± 1.15% and 0.73 ± 0.83%, respectively), while decanal and dodecanal were lower in FHC1 (1.30 ± 3.26% and 0.04 ± 0.03%, respectively) than in FHC2 (4.04 ± 7.08% and 0.58 ± 1.58%, respectively). The percentage composition of *β*-phellandrene in DHC1 (0.99 ± 2.02%) was higher than in DHC2 (0.03 ± 0.05%), whereas phytol was lower in DHC1 (0.54 ± 0.20%) than in DHC2 (1.63 ± 1.11%). These differences between the samples might be attributed to the medicinal parts, drying process, and collection seasons [1, 3]. In order to further explore the distinctions between DHC and FHC and their two harvest seasons, chemometric methods were utilized.

### 3.3. Heatmap and HCA and PCA Analysis

**Figure 3:**
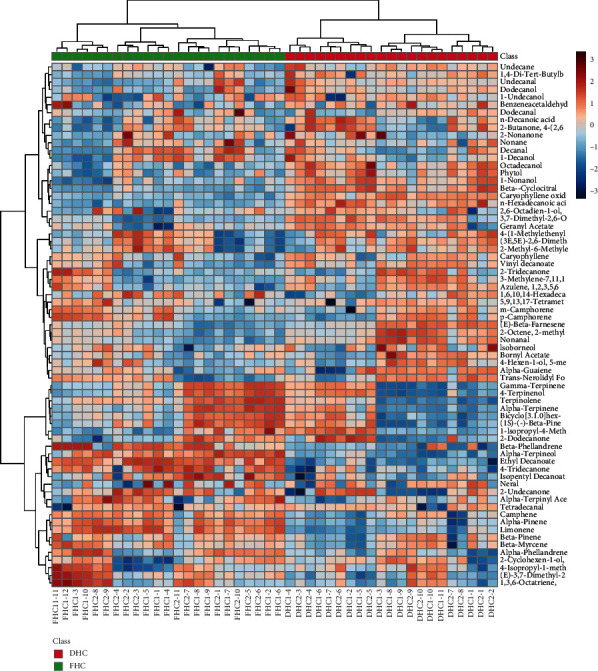
Heatmap analysis and HCA analysis of twenty-one batches of DHC and twenty-three batches of FHC at two harvest seasons. The different degrees of color clearly indicate the relationship between these chemical components in different samples.

To make the overall discrepancies in the chemical profiles of DHC and FHC oils more externally, heatmap and HCA analysis were further performed to explore the prominent distinctions between DHC and FHC. Heatmap is one of the most popular bioinformatic graphic displays for data visualization by color intensity. HCA is a clustering technique that measures either the difference or the similarity among the samples to be clustered. Based on the relative contents of each constituent in the EOs of the samples, the position of various samples will be crudely redistributed, and samples with close similarities will be classified into the same group by HCA. As illustrated in Figure 3, the blue box indicated that the content was lower, while the red box showed a greater content than the average level of the sample. It was noted that forty-four batches of samples were apparently classified into two categories; i.e., the first group included twenty-one batches of DHC and the second group twenty-three batches of FHC. Nevertheless, eleven batches of DHC1 and ten batches of DHC2 could not be well clustered; neither were twelve batches of FHC1 nor eleven batches of FHC2. In other words, although there was significant difference in chemical composition between DHC and FHC, the difference between DHC1 and DHC2 and FHC1 and FHC2 was not obvious.

**Figure 4:**
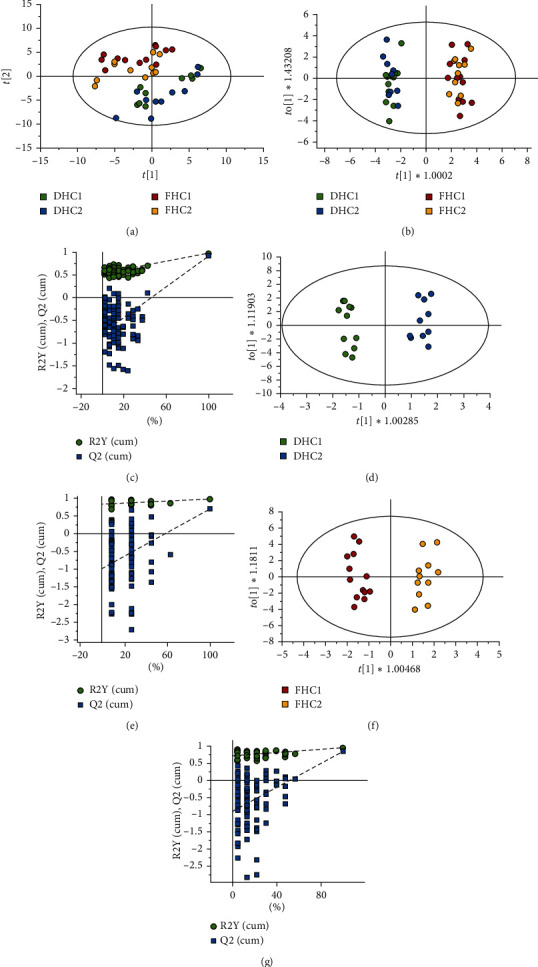
PCA analysis and OPLS-DA analysis of DHC and FHC and their two harvest seasons. PCA score plot for DHC and FHC (a). OPLS-DA score plot and the corresponding validation plots based on 200 times permutation tests of the OPLS-DA model for DHC and FHC (b, c), for DHC1 and DHC2 (d, e), and for FHC1 and FHC2 (f, g).

Moreover, another unsupervised PCA approach was employed to provide more information about the discrimination of DHC and FHC (Figure 4(a)). The results showed that R^2^X = 0.773 and *Q*^2^ = 0.519, which indicated that the model was reliable and had good predictive ability. The score plot showed that the DHC (both blue and green spots) and FHC (both red and yellow spots) were clearly separated. However, DHC1 (green spots) and DHC2 (blue spots) and FHC1 (red spots) and FHC2 (yellow spots) could not be classified obviously. The result was consistent with heatmap and HCA analysis. In general, HCA and PCA are both good classification tools that distinguished DHC and FHC. Still, they could not well classify the samples at two harvest seasons and could not find out the major chemical differential components between them. To find their chemical markers for discrimination, OPLS-DA analysis was applied.

### 3.4. OPLS-DA Analysis and Identification of Chemical Markers

To further distinguish the major variations responsible for the differentiation and find the chemical markers, a supervised OPLS-DA approach was constructed to maximize sample separation. As indicated in Figure 4(b), the samples of DHC and FHC were distributed on two sides evidently. The variation in X (R2Xcum) was 0.658 and the variation in Y (R2Ycum) was 0.975, predicting 91.9% of the variation in response to Y (Q2cum = 0.919). The value of these parameters demonstrated that the model had high reliability and predictive abilities. To validate the model, 200 times permutation tests were performed (Figure 4(c)). Moreover, the *p* value and VIP value of the 69 common constituents were obtained. The constituents with VIP value >1.5 and *p* value <0.01 were regarded as potential chemical markers responsible for the distinctions. As a result, the eleven components including *α*-pinene, limonene, *β*-phellandrene, *α*-terpineol, 4-tridecanone, ethyl decanoate, nonanal, 1-nonanol, *β*-cyclocitral, n-hexadecanoic acid, and octadecanol had significant qualitative differences, which were screened as chemical markers between DHC and FHC.

Regarding the two harvest seasons of DHC, the samples of DHC1 and DHC2 were clearly classified and aggregated on the scatter plot (Figure 4(d)). The variation in X (R2Xcum) was 0.617 and the variation in Y (R2Ycum) was 0.974, predicting 70.4% of the variation in response to Y (Q2cum = 0.704). Using the same screening criteria as used above, two components, including 4-tridecanone and ethyl decanoate, were identified as chemical markers for DHC1 and DHC2. In addition, the samples of FHC1 and FHC2 were distributed on two sides obviously, as indicated in Figure 4(f). The variation in X (R2Xcum) was 0.637 and the variation in Y (R2Ycum) was 0.956, predicting 83.3% of the variation in response to Y (Q2cum = 0.833). Four components, including *β*-myrcene, *β*-phellandrene, 2,6-octadien-1-ol,3,7-dimethyl-acetate, and (z)-phytol, were identified as chemical markers for FHC1 and FHC2. All the chemical markers are shown in Table 2.

### 3.5. Content Comparison of Chemical Markers and Their Pharmacological Effects

Given that the EO of HC exerts various pharmacological activities [2, 3], we explored whether the above chemical markers possess these activities. As present in Table 2, five chemical markers (*α*-pinene, limonene, *β*-phellandrene,*α*-terpineol, and n-hexadecanoic acid) between DHC and FHC and three chemical markers (*β*-myrcene, *β*-phellandrene, and phytol) in FHC at two harvest seasons exhibit a wide range of pharmacological activities including anti-inflammatory, ant-oxidant, antiallergy, antibacterial, antiviral, immunologic, anticancer, antianxiety, analgesic, and antidiabetic effects [13–31]. Therefore, the different contents of these chemical markers in DHC and FHC and the two harvest seasons of FHC might influence the quality and efficacy.

**Figure 5:**
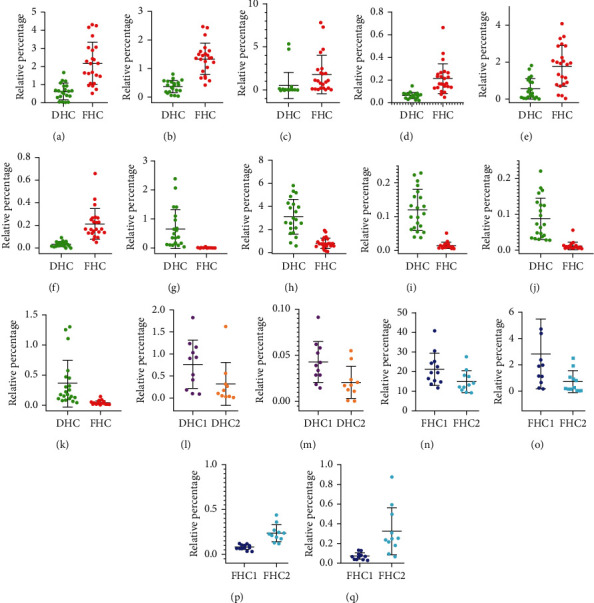
Relative content of chemical markers of DHC and FHC (a–k), DHC1 and DHC2 (l, m), and FHC1 and FHC2 (n–q). (a) *α*-Pinene; (b) limonene; (c) *β*-phellandrene; (d) *α*-terpineol; (e) 4-tridecanone; (f) ethyl decanoate; (g) nonanal; (h) 1-nonanol; (i) *β*-cyclocitral; (j) n-hexadecanoic acid; (k) octadecanol; (l) 4-tridecanone; (m) ethyl decanoate; (n) *β*-myrcene; (o) *β*-phellandrene; (p) 2,6-octadien-1-ol, 3,7-dimethyl-, acetate, (z)-; (q) phytol.

As shown in Figure 5, scatter plots were applied to further intuitively compare the content differences of the chemical markers in the three groups (DHC and FHC; DHC1 and DHC2 FHC1 and FHC2). Comparing DHC and FHC, the average contents of *α*-pinene^a^ (0.63 ± 0.44% and 2.18 ± 1.15%, respectively), limonene^b^ (0.37 ± 0.22% and 1.35 ± 0.55%, respectively), *β*-phellandrene^c^ (0.53 ± 1.51% and 1.82 ± 2.23%, respectively), *α*-terpineol^d^ (0.06 ± 0.03% and 0.21 ± 0.13%, respectively), 4-tridecanone^e^ (0.55 ± 0.56% and 1.78 ± 1.11%, respectively), and ethyl decanoate^f^ (0.03 ± 0.02% and 0.80 ± 0.46%, respectively) were higher in FHC oils, while the average contents of nonanal^g^ (0.67 ± 0.68% and 0.07 ± 0.04%, respectively), 1-nonanol^h^ (3.10 ± 1.50% and 0.49 ± 0.55%, respectively), *β*-cyclocitral^i^ (0.12 ± 0.06% and 0.01 ± 0.01%, respectively), n-hexadecanoic acid^j^ (0.09 ± 0.06%, and 0.01 ± 0.01%, respectively), and octadecanol^k^ (0.36 ± 0.39% and 0.04 ± 0.03%, respectively) from DHC oils were higher than those from FHC oils. Among these markers, *α*-pinene^a^, limonene^b^, *β*-phellandrene^c^, and *α*-terpineol^d^ higher in FHC had several pharmacological activities, especially anti-inflammatory [13, 15], antioxidant [14, 19], antimicrobial [20], and anticancer activities [14, 20, 21]. Meanwhile, only one chemical marker, *n*-hexadecanoic acid^j^, with anti-inflammatory activity [26] was higher in DHC. This result suggested that FHC should be considered as the first choice when it was used as an anti-inflammatory, antioxidant, anticancer, and antibacterial herb. The suggestion was consistent with previous reports [1]. Additionally, the contents of 4-tridecanone^l^ (0.76 ± 0.55% and 0.32 ± 0.49%, respectively) and ethyl decanoate^m^ (0.04 ± 0.02% and 0.02 ± 0.02%, respectively) were higher in DHC1 oils than DHC2 oils. The contents of *β*-myrcene^n^ (21.33 ± 8.72% and 15.12 ± 5.58%, respectively) and *β*-phellandrene^o^ (2.82 ± 2.65% and 0.73 ± 0.83%, respectively) higher in FHC1 oils had antitumor [21] and antioxidant activities [22, 27], while the contents of 2,6-octadien-1-ol, 3,7-dimethyl-, acetate^p^ (0.08 ± 0.03% and0.23 ± 0.09%, respectively), and (z)-phytol^q^ (0.07 ± 0.03% and 0.33 ± 0.24%, respectively) with anti-inflammatory and antibacterial activities [29, 30] were higher in FHC2 oils.

## 4. Conclusions

In this work, GS-MS analysis and chemometric methods were applied to compare the oils of DHC and FHC in two harvest seasons. The results showed that EO yields of FHC were higher than DHC, and EO yields of DHC1/FHC1 collected in summer were higher than of DHC2/FHC2 collected in autumn. GC-MS fingerprints showed that DHC and FHC were similar in general and 69 common chemical constituents were characterized. 2-Undecanone, *β*-myrcene, and *β*-pinene (accounting for about 70% of the total) were the three most abundant components in both DHC and FHC. Nevertheless, the contents of some constituents were significantly different such as *α*-pinene, *β*-phellandrene, 1-nonanol, and geranyl acetate. Moreover, the results of chemometric analysis including HCA, PCA, and OPLS-DA indicated obvious distinction between DHC and FHC and the two harvest seasons of DHC (DHC1 and DHC2) and FHC (FHC1 and FHC2), and OPLS-DA further revealed 11, 2, and 4 components as their potential chemical markers, respectively. Through this study, we found that the processing methods (i.e., medicinal parts and drying process) and harvest seasons can directly affect the chemical composition of HC and their quality. Future studies are needed to verify whether the differences between them would influence the pharmacological effects to provide a better reference for clinical medication.

## Figures and Tables

**Table 1:** Average relative content of sixty-nine common volatile constituents of DHC and FHC at two harvest seasons.

No.	Name/class	RT (min)	Similarity (%)	Average relative content (%)					
				DHC (*n* = 21)	FHC (*n* = 23)	DHC1 (*n* = 11)	DHC2 (*n* = 10)	FHC1 (*n* = 12)	FHC2 (*n* = 11)
*Aliphatic compounds*									
1	Nonane	6.47	99	0.02 (0.02)	0.02 (0.03)	0.02 (0.01)	0.03 (0.03)	0.02 (0.03)	0.02 (0.02)
2	2-Nonanone	10.38	96	0.06 (0.10)	0.02 (0.02)	0.09 (0.13)	0.02 (0.01)	0.01 (0.01)	0.03 (0.03)
3	Undecane	10.59	98	0.04 (0.03)	0.02 (0.01)	0.04 (0.04)	0.04 (0.03)	0.01 (0.01)	0.02 (0.01)
4	Nonanal	10.75	90	0.67 (0.68)	0.07 (0.04)	0.81 (0.85)	0.50 (0.39)	0.05 (0.03)	0.08 (0.05)
5	1-Nonanol	12.75	99	3.10 (1.50)	0.49 (0.55)	2.73 (1.15)	3.51 (1.80)	0.31 (0.21)	0.69 (0.73)
6	Decanal	13.88	99	2.68 (6.35)	2.61 (5.48)	2.55 (7.32)	2.82 (5.47)	1.30 (3.26)	4.04 (7.08)
7	1-Decanol	15.53	99	0.22 (0.55)	0.14 (0.20)	0.29 (0.75)	0.13 (0.13)	0.07 (0.08)	0.22 (0.27)
8	2-Undecanone	16.25	92	44.92 (27.15)	48.49 (15.48)	44.23 (30.29)	45.67 (24.73)	45.94 (13.18)	51.27 (17.87)
9	Undecanal	16.5	99	0.19 (0.40)	0.09 (0.22)	0.25 (0.54)	0.14 (0.16)	0.02 (0.04)	0.17 (0.31)
10	1-Undecanol	18.31	99	0.60 (0.83)	0.17 (0.22)	0.68 (0.97)	0.52 (0.70)	0.17 (0.12)	0.18 (0.30)
11	2-Dodecanone	19.01	91	0.30 (0.31)	0.22 (0.17)	0.28 (0.28)	0.31 (0.36)	0.17 (0.12)	0.28 (0.21)
12	*n*-Decanoic acid	19.02	98	0.08 (0.05)	0.04 (0.03)	0.06 (0.03)	0.10 (0.07)	0.02 (0.01)	0.05 (0.03)
13	Dodecanal	19.48	99	0.12 (0.17)	0.30 (1.10)	0.07 (0.06)	0.17 (0.23)	0.04 (0.03)	0.58 (1.58)
14	Dodecanol	21.76	99	0.10 (0.19)	0.04 (0.07)	0.12 (0.26)	0.07 (0.04)	0.02 (0.02)	0.06 (0.09)
15	Vinyl decanoate	21.8	89	0.10 (0.05)	0.05 (0.03)	0.08 (0.04)	0.11 (0.05)	0.04 (0.01)	0.06 (0.03)
16	4-Tridecanone	22.66	89	0.55 (0.56)	1.78 (1.11)	0.76 (0.55)	0.32 (0.49)	2.03 (0.79)	1.52 (1.37)
17	2-Tridecanone	22.67	92	2.29 (2.16)	1.18 (1.10)	2.58 (2.20)	1.96 (2.20)	1.41 (1.41)	0.92 (0.58)
18	Ethyl decanoate	27.81	82	0.03 (0.02)	0.80 (0.46)	0.04 (0.02)	0.02 (0.02)	0.81 (0.26)	0.79 (0.63)
19	Tetradecanal	28.4	99	1.48 (1.19)	0.82 (0.38)	1.27 (1.12)	1.72 (1.27)	0.78 (0.39)	0.87 (0.37)
20	Isopentyl decanoate	36.36	78	0.06 (0.04)	0.11 (0.07)	0.06 (0.02)	0.06 (0.06)	0.09 (0.05)	0.13 (0.09)
21	*n*-Hexadecanoic acid	42.62	94	0.09 (0.06)	0.01 (0.01)	0.07 (0.03)	0.11 (0.07)	0.01 (0.00)	0.02 (0.01)
22	Octadecanol	45.57	99	0.36 (0.39)	0.04(0.03)	0.16 (0.09)	0.58 (0.48)	0.02 (0.01)	0.05 (0.04)

*Aromatic compounds*									
23	Benzeneacetaldehyde	9.35	92	0.06 (0.05)	0.04 (0.04)	0.05 (0.03)	0.08 (0.06)	0.04 (0.05)	0.03 (0.02)
24	4-Isopropyl-1-methylcyclohex-2-enol	11.55	96	0.08 (0.10)	0.06 (0.08)	0.07 (0.06)	0.10 (0.13)	0.08 (0.10)	0.05 (0.05)
25	2-Butanone, 4-(2,6,6-trimethyl-1,3-cyclohexadien-1-yl)-	19.96	85	0.05 (0.05)	0.02 (0.02)	0.03 (0.02)	0.08 (0.06)	0.01 (0.01)	0.03 (0.02)
26	1,4-Di-Tert-Butylbenzene	21.75	80	0.10 (0.12)	0.03 (0.04)	0.11 (0.17)	0.08 (0.05)	0.02 (0.01)	0.05 (0.06)

*Monoterpene hydrocarbons*									
27	Bicyclo[3.1.0]hex-2-ene,2-methyl-5-(1-methylethyl)-	7.04	92	0.04 (0.05)	0.06 (0.06)	0.03 (0.04)	0.05 (0.06)	0.05 (0.06)	0.07 (0.07)
28	*α*-Pinene	7.23	99	0.63 (0.44)	2.18 (1.15)	0.66 (0.43)	0.59 (0.48)	2.24 (1.14)	2.11 (1.22)
29	Camphene	7.54	99	0.18 (0.14)	0.26 (0.08)	0.20 (0.16)	0.15 (0.13)	0.28 (0.08)	0.25 (0.09)
30	(1S)-(-)-*β*-Pinene	7.92	91	0.77 (1.05)	1.64 (1.79)	0.62 (0.95)	0.95 (1.17)	1.62 (1.97)	1.67 (1.65)
31	*β*-Pinene	8.09	99	9.95 (7.16)	8.67 (3.43)	10.59 (7.40)	9.25 (7.21)	9.23 (3.93)	8.07 (2.85)
32	*β*-Myrcene	8.14	99	17.68 (12.99)	18.36 (7.64)	18.85 (13.44)	16.39 (13.06)	21.33 (8.72)	15.12 (5.58)
33	*α*-Phellandrene	8.54	98	0.02 (0.02)	0.03 (0.02)	0.02 (0.02)	0.02 (0.01)	0.03 (0.01)	0.04 (0.03)
34	*α*-Terpinene	8.78	99	0.06 (0.08)	0.16 (0.17)	0.05 (0.07)	0.08 (0.08)	0.13 (0.14)	0.19 (0.20)
35	1-Isopropyl-4-Methylbenzene	8.94	95	0.09 (0.10)	0.08 (0.13)	0.09 (0.11)	0.09 (0.10)	0.06 (0.13)	0.10 (0.14)
36	Limonene	9.06	98	0.37 (0.22)	1.35 (0.55)	0.36 (0.20)	0.38 (0.24)	1.31 (0.52)	1.38 (0.60)
37	*β*-Phellandrene	9.13	93	0.53 (1.51)	1.82 (2.23)	0.99 (2.02)	0.03 (0.05)	2.82 (2.65)	0.73 (0.83)
38	1,3,6-Octatriene, 3,7-dimethyl-, (Z)-	9.31	96	0.07 (0.06)	0.10 (0.09)	0.06 (0.05)	0.08 (0.07)	0.11 (0.10)	0.09 (0.07)
39	2-Octene, 2-methyl-6-methylene-	9.47	80	0.08 (0.07)	0.01 (0.01)	0.09 (0.09)	0.07 (0.05)	0.01 (0.01)	0.02 (0.01)
40	Gamma-terpinene	9.73	99	0.22 (0.28)	0.44 (0.47)	0.19 (0.27)	0.25 (0.29)	0.32 (0.36)	0.56 (0.56)
41	Terpinolene	10.54	96	0.05 (0.04)	0.11 (0.08)	0.05 (0.04)	0.05 (0.04)	0.09 (0.07)	0.14 (0.10)

*Sesquiterpene hydrocarbons*									
42	Caryophyllene	20.56	99	0.27 (0.13)	0.16 (0.08)	0.23 (0.10)	0.31 (0.15)	0.13 (0.04)	0.19 (0.11)
43	*α*-Guaiene	22.37	91	0.07 (0.05)	0.03 (0.01)	0.07 (0.05)	0.08 (0.05)	0.03 (0.01)	0.03 (0.02)
44	Azulene, 1,2,3,5,6,7,8,8a-octahydro-1,4-dimethyl-7-(1-methylethenyl)-, [1S-(1.*α*.,7.*α*.,8a.*β*.)]-	23.5	91	0.14 (0.08)	0.07 (0.04)	0.13 (0.07)	0.16 (0.080	0.07 (0.05)	0.07 (0.03)

*Diterpene hydrocarbons*									
45	3-Methylene-7,11,15-Trimethyl-1,6,10,14-Hexadecatetraene	21.19	90	0.19 (0.12)	0.07 (0.04)	0.17 (0.0)	0.21 (0.13)	0.07 (0.04)	0.07 (0.03)
46	m-Camphorene	42.42	95	0.19 (0.18)	0.11 (0.08)	0.19 (0.15)	0.19 (0.21)	0.12 (0.09)	0.09 (0.06)
47	p-Camphorene	43.3	94	0.09 (0.09)	0.05 (0.04)	0.08 (0.07)	0.10 (0.11)	0.06 (0.05)	0.04 (0.04)
48	(E)-*β*-Farnesene	46.1	78	0.26 (0.27)	0.03 (0.02)	0.22 (0.20)	0.29 (0.33)	0.02 (0.01)	0.04 (0.02)

*Oxygenated monoterpenes*									
49	(E)-3,7-Dimethyl-2,6-octadien-1-ol	9.06	90	1.55 (1.73)	2.44 (1.99)	1.59 (1.78)	1.51 (1.78)	3.04 (2.44)	1.80 (1.15)
50	(3E,5 E)-2,6-Dimethylocta-3,5,7-trien-2-ol	10.62	85	0.07 (0.04)	0.03 (0.02)	0.06 (0.04)	0.09 (0.05)	0.03 (0.02)	0.04 (0.03)
51	2-Methyl-6-Methylene-2,7-octadien-4-ol	12.06	92	0.07 (0.05)	0.04 (0.03)	0.06 (0.04)	0.08 (0.06)	0.03 (0.02)	0.05 (0.03)
52	Isoborneol	13.03	96	0.09 (0.07)	0.05 (0.02)	0.10 (0.07)	0.07 (0.06)	0.04 (0.01)	0.06 (0.02)
53	4-Terpinenol	13.31	99	0.52 (0.55)	0.93 (1.06)	0.53 (0.61)	0.51 (0.51)	0.65 (0.77)	1.25 (1.26)
54	Neral	13.45	87	0.02 (0.02)	0.02 (0.03)	0.02(0.01)	0.02 (0.030	0.02 (0.03)	0.01 (0.01)
55	*α*-Terpineol	13.63	99	0.06 (0.03)	0.21 (0.13)	0.07 (0.03)	0.06 (0.02)	0.17 (0.09)	0.26 (0.16)
56	2-Cyclohexen-1-ol, 3-methyl-6-(1-methylethyl)-, trans-	14.06	91	0.06 (0.04)	0.03 (0.02)	0.06 (0.05)	0.05 (0.04)	0.03 (0.02)	0.04 (0.03)
57	*β*-Cyclocitral	14.36	91	0.12 (0.06)	0.01 (0.01)	0.09 (0.05)	0.15 (0.06)	0.01 (0.00)	0.02 (0.01)
58	3,7-Dimethyl-2,6-octadien-1-ol(trans)	15.1	99	0.18 (0.11)	0.03 (0.01)	0.18 (0.09)	0.17 (0.13)	0.02 (0.01)	0.03 (0.02)
59	4-(1-Methylethenyl)cyclohexene-1-methanol	15.33	86	0.08 (0.06)	0.04 (0.03)	0.05 (0.03)	0.12 (0.07)	0.03 (0.02)	0.05 (0.03)
60	Bornyl acetate	16.18	92	1.89 (0.92)	1.28 (0.26)	1.85 (1.06)	1.93 (0.80)	1.27 (0.22)	1.30 (0.31)
61	*α*-Terpinyl acetate	17.86	85	0.06 (0.03)	0.07 (0.04)	0.05 (0.03)	0.06 (0.03)	0.06 (0.04)	0.08 (0.04)
62	4-Hexen-1-ol, 5-methyl-2-(1-methylethenyl)-, acetate	18.02	90	0.11 (0.08)	0.06 (0.03)	0.09 (0.07)	0.12 (0.08)	0.04 (0.01)	0.07 (0.04)
63	Geranyl acetate	18.62	98	2.65 (1.80)	0.75 (0.46)	2.23 (0.94)	3.12 (2.41)	0.59 (0.30)	0.91 (0.56)
64	2,6-Octadien-1-ol, 3,7-dimethyl-, acetate, (Z)-	18.63	94	0.28 (0.21)	0.15 (0.10)	0.22 (0.12)	0.34 (0.27)	0.08 (0.03)	0.23 (0.09)

*Oxygenated sesquiterpenes*									
65	*trans*-Nerolidyl formate	26.01	97	0.34 (0.35)	0.07 (0.06)	0.32 (0.27)	0.36 (0.450	0.07 (0.05)	0.07 (0.06)
66	Caryophyllene oxide	27.95	96	0.37 (0.25)	0.04 (0.02)	0.34 (0.25)	0.39 (0.27)	0.03 (0.01)	0.05 (0.03)
	Oxygenated diterpenes								
67	5,9,13,17-Tetramethyl 4,8,12,16-Octadecatetraenoic acid	34.51	88	0.08 (0.11)	0.03 (0.04)	0.06 (0.05)	0.11 (0.15).	0.02 (0.01)	0.04 (0.06)
68	Phytol	46.42	96	1.06 (0.94)	0.19 (0.21)	0.54 (0.20)	1.63 (1.11)	0.07 (0.03)	0.33 (0.24)
69	1,6,10,14-Hexadecatetraen-3-ol,3,7,11,15-tetramethyl-, (E,E)-	56.29	81	0.07 (0.05)	0.06 (0.03)	0.05 (0.03)	0.08 (0.07)	0.04 (0.01)	0.08 (0.03)
	Total identified (%)			95.62 (4.63)	98.41 (2.01)	94.88 (5.12)	96.43 (4.13)	99.09 (0.61)	97.67 (2.70)
*Class composition*									
Nonterpene compounds				58.34 (23.28)	57.66 (12.39)	57.51 (25.68)	59.25(21.67)	53.51 (12.04)	62.19 (11.62)
Aliphatic compounds				58.04 (23.34)	57.50 (12.47)	57.25 (25.74)	58.91 (21.75)	53.36 (12.15)	62.03 (11.67)
Aromatic compounds				0.30 (0.18)	0.15 (0.11)	0.25 (0.18)	0.34 (0.17)	0.15 (0.13)	0.16 (0.09)
Terpenoids				41.66 (23.28)	42.34 (12.39)	42.49 (25.68)	40.75 (21.67)	46.49 (12.04)	37.81 (11.62)
Terpene hydrocarbons				31.95 (21.79)	35.79 (10.95)	33.93 (23.15)	29.77 (21.20)	40.13 (10.06)	31.05 (10.25)
Monoterpene hydrocarbons				30.74 (21.40)	35.27 (10.85)	32.84 (22.53)	28.42 (21.04)	39.62 (9.88)	30.52 (10.21)
Sesquiterpene hydrocarbons				0.48 (0.22)	0.26 (0.11)	0.42 (0.19)	0.55 (0.24)	0.23 (0.08)	0.29 (0.13)
Diterpene hydrocarbons				0.73 (0.62)	0.26 (0.17)	0.66 (0.51)	0.80 (0.75)	0.28 (0.19)	0.24 (0.14)
Oxygenated terpenes				9.72 (4.07)	6.55 (2.09)	8.56 (3.21)	10.99 (4.69)	6.36 (2.33)	6.76 (1.88)
Oil yield (%, v/w)				0.04 (0.03)	0.08 (0.03)	0.04 (0.03)	0.03 (0.03)	0.08 (0.03)	0.07 (0.03)
Total identified (%)				95.62 (4.63)	98.41 (2.01)	94.88 (5.12)	96.43 (4.13)	99.09 (0.61)	97.67 (2.70)

Note: RT represents retention time. Data were presented as mean (SD).

**Table 2:** Pharmacological effects of chemical markers identified for DHC and FHC, DHC1 and DHC2, and FHC1 and FHC2 from the OPLS-DA validation plot.

Compound		Chemical formula	Classification	*p* value	VIP value	Pharmacological effects
DHC- FHC	*α*-Pinene^a^	C_10_H_16_	Monoterpene hydrocarbons	7.37 × 10^−8^	1.70	Anti-inflammatory [13], antioxidant [14], antitumor [14], antifungal [14], antiallergy, improving ulcer [14], antianxiety [14]
	Limonene^b^	C_10_H_16_	Monoterpene hydrocarbons	8.43 × 10^−11^	1.76	Anti-inflammatory [15], antidiabetic effects [16], immunomodulatory activity [17], analgesic [18], hypolipidemic and antioxidant activities [19], antimicrobial activity [20], anticancer activity [20], insecticidal activity [20]
	*β*-Phellandrene^c^	C_10_H_16_	Monoterpene hydrocarbons	8.75 × 10^−7^	2.19	Antitumor activity [21], antioxidant activities [22]
	*α*-Terpineol^d^	C_10_H_18_O	Oxygenated monoterpenes	5.04 × 10^−12^	1.60	Anti-inflammatory and analgesic [23], anticonvulsant [24], gastric protection effects [25]
	4-Tridecanone^e^	C_13_H_26_O	Aliphatic compounds	7.85 × 10^−5^	1.53	—
	Ethyl decanoate^f^	C_12_H_24_O_2_	Aliphatic compounds	1.23 × 10^−16^	2.68	—
	Nonanal^g^	C_9_H_18_O	Aliphatic compounds	1.03 × 10^−7^	1.54	—
	1-Nonanol^h^	C_9_H_20_O	Aliphatic compounds	8.5810^–9^	1.68	—
	*β*-Cyclocitral^i^	C_10_H_16_O	Oxygenated monoterpenes	2.07 × 10^−12^	1.78	—
	*n*-Hexadecanoic acid^j^	C_16_H_32_O_2_	Aliphatic compounds	2.11 × 10^−11^	1.67	Anti-inflammatory [26]
	Octadecanol^k^	C_18_H_38_O	Aliphatic compounds	3.58 × 10^−8^	1.68	—

DHC1-DHC2	4-Tridecanone^l^	C_13_H_26_O	Aliphatic compounds	3.43 × 10^−4^	3.34	—
	Ethyl decanoate^m^	C_15_H_24_O	Oxygenated sesquiterpenes	2.39 × 10^−3^	2.56	—

FHC1-FHC2	*β*-Myrcene^n^	C_10_H_16_	Monoterpene hydrocarbons	4.70 × 10^−6^	1.67	Antioxidant [27], liver monooxygenase induction effects [28]
	*β*-Phellandrene^o^	C_10_H_16_	Monoterpene hydrocarbons	7.60 × 10^−3^	2.56	Antitumor activity [21], antioxidant activities [22]
	2,6-Octadien-1-ol, 3,7-dimethyl-, acetate, (Z)-^p^	C_12_H_20_O_2_	Oxygenated monoterpenes	5.29 × 10^−5^	2.15	—
	Phytol^q^	C_20_H_40_O	Oxygenated diterpenes	1.27 × 10^−3^	2.32	Anti-inflammatory [29], antibacterial [30], antischistosomiasis [31]

Note: VIP > 1.5 and *p* < 0.01.The symbol “-” stands for none.

## Data Availability

The data used to support the finding of this study are included within the article.
